# Streptozotocin-Induced Diabetic Rats Showed a Differential Glycine Receptor Expression in the Spinal Cord: A GlyR Role in Diabetic Neuropathy

**DOI:** 10.1007/s11064-023-04058-9

**Published:** 2023-11-29

**Authors:** Miguel Ángel Velázquez-Flores, Gustavo Sánchez-Chávez, Sara L. Morales-Lázaro, Ruth Ruiz Esparza-Garrido, Alejandro Canizales-Ontiveros, Rocío Salceda

**Affiliations:** 1 Noncoding RNAs Laboratory, Unit of Medical Research on Human Genetics, Children’s Hospital “Silvestre Frenk Freund”, National Medical Center Century XXI, Mexican Institute of Social Security, Mexico City, Mexico; 2 División de Neurociencias, Instituto de Fisiología Celular, Universidad Nacional Autónoma de México, Mexico City, Mexico; 3 Unit of Medical Research on Human Genetics, Children’s Hospital “Silvestre Frenk Freund”, National Medical Center Century XXI, Mexican Institute of Social Security, Mexico City, Mexico

**Keywords:** Glycine receptor, Diabetes, Diabetic neuropathy, Spinal cord

## Abstract

In the spinal cord, attenuation of the inhibitory action of glycine is related to an increase in both inflammatory and diabetic neuropathic pain; however, the glycine receptor involvement in diabetic neuropathy has not been reported. We determined the expression of the glycine receptor subunits (α1–α3 and β) in streptozotocin-induced diabetic Long–Evans rats by qPCR and Western blot. The total mRNA and protein expression (whole spinal cord homogenate) of the α1, α3, and β subunits did not change during diabetes; however, the α2 subunit mRNA, but not the protein, was overexpressed 45 days after diabetes induction. By contrast, the synaptic expression of the α1 and α2 subunits decreased in all the studied stages of diabetes, but that of the α3 subunit increased on day 45 after diabetes induction. Intradermal capsaicin produced higher paw-licking behavior in the streptozotocin-induced diabetic rats than in the control animals. In addition, the nocifensive response was higher at 45 days than at 20 days. During diabetes, the expression of the glycine receptor was altered in the spinal cord, which strongly suggests its involvement in diabetic neuropathy.

**Supplementary Information:**

The online version contains supplementary material available at 10.1007/s11064-023-04058-9.

## Introduction

Diabetes is the most common cause of peripheral neuropathy occurring in 70–90% of patients with diabetes, and it is frequently associated with severe neuropathic pain [1, 2]. Strict control of glucose levels is enough to reduce the intensity of pain and to prevent further deterioration in diabetic patients [3]. Hyperactivity of spinal dorsal horn neurons plays an important role in the development of diabetic neuropathic pain. Glycine is the main inhibitory neurotransmitter in the spinal cord, and evidence indicates its involvement in pain sensitization [4, 5]. In the dorsal horn, the attenuation of glycinergic neurotransmission by decreasing glycine release [6] or by blocking glycine receptors (GlyRs) with strychnine [7–10] can elicit tactile allodynia, a major symptom of neuropathic pain. By contrast, the activation of these neurons alleviated neuropathic hyperalgesia and itch [5, 7, 11]. Similarly, the decrease in the function of α3/β GlyRs, by PKA-dependent phosphorylation in response to prostaglandin E2 (PGE2) action also induced hyperalgesia and allodynia [12, 13]. These data indicate that the attenuation of the inhibitory action of glycine is associated with pain sensitization. Despite this information, there is little information related to changes in the synaptic input to spinal dorsal horn neurons in diabetic neuropathy. Therefore, we evaluated the α1–α3 and β GlyR subunit expression in the rat spinal cord shortly after diabetes was induced with streptozotocin (STZ).

## Methods

### Animals

Adult Long–Evans rats (150–200 g) were used for the experiments; they were randomly divided in control and diabetic groups. Diabetes was induced by a single intraperitoneal STZ administration (90 mg/k, ip.) in buffer citrate, pH 4.5 [14]. Blood glucose levels from the tail-vein blood samples were measured using ACCU-CHEK test strips (Roche Diagnostics), 24–48 h after STZ administration and at sacrifice. Age-matched citrate buffer-injected rats were used as the control group. Rats were maintained (4–5 per cage) at 21 °C ± 1, 12 h light–dark cycle, and food and water provided ad libitum. Diabetes was confirmed by measuring blood glucose concentration and loss of gain body weight (Table 2). Animals were considered diabetic if blood glucose levels were higher than 250 mg/dl; insulin was not administered.

Previous studies demonstrated that lower STZ doses (60 mg/kg) has an efficacy of 60% in the induction of the hyperglycemic condition, while higher doses (90 mg/kg) have an efficacy of 95%. These studies also indicated that 12–15% of males did not became hyperglycemic after STZ treatment, while in only 2–5% of females we observed this STZ resistance. Therefore, in the present study, we used the relative high STZ doses and female rats. Animals were sacrificed at 7, 20, and 45 days after diabetes induction (7D; 20D; and 45D), along with non-treated animals (controls). A different set of control and STZ treated rats were used for biochemical or licking behavior. The lumbar spinal cord (L5 and L6, 0.2–0.25 g) was isolated for mRNA and Western blots (WB) assays.

### Ethics

This study was conducted in strict accordance with the recommendations of the Mexican Institutes of Health Research (DOF. NOM-062-Z00-1999). The protocol was approved by the Institutional Laboratory Animal Care and Use Committee of the Cellular Physiology Institute of the National Autonomous University of Mexico (CICUAL, Comité Institucional para el Cuidado y Uso de los Animales de Laboratorio del Instituto de Fisiología Celular de la Universidad Nacional Autónoma de México). Protocol number: RSS190-22 and RSS110 (43)-17. All efforts were made to minimize animal suffering and to reduce the number of rats used.

### Nocifensive Test

The capsaicin-evoked nocifensive response was evaluated in non-treated animals (control) and STZ-induced diabetic rats at 20 days and 45 days [15]. The animals were placed in individual plastic containers 1 h before the experiment. The stock solution of capsaicin was resuspended in ethanol (10 µg/µl) (Sigma-Aldrich). The injection and vehicle solutions were respectively prepared by diluting capsaicin (0.19 µg/µl) in saline solution (0.19 µg/µl) and in 1.9% ethanol. Both, control and diabetic rats were first intraplantarly injected on the left paw with 10 µl of saline solution, using a 30 G needle; then, the animals were placed in the containers and the licking behavior was quantified for 10 min. After 30 min adaptation, rats were intraplantarly injected on the right paw with 10 µl capsaicin solution, were placed in the containers, and the licking behavior was quantified for 10 min. The cumulative licking time (seconds) was reported as paw licking time (PLT).

### Synaptosomes Preparation

Lumbar spinal cord was dissected and synaptosomes were isolated by the procedure described by Hajos [16] and slightly modified by Pérez-Léon and Salceda [17]. Tissue was homogenized in 0.3 M sucrose (10% w/v)—Tris 10 mM, pH 7.4 and centrifuged at 1500×*g* for 10 min. The supernatant was centrifuged at 9000×*g* for 20 min. The obtained pellet (crude synaptosomal fraction) was used for Western blot or qPCR.

### RNA Extraction

Total RNA was extracted with TRIZOL (Ambion Life Technologies, Thermo Scientific Inc.), as previously described by [18]. cDNA was synthesized with the RevertAid H Minus First Strand cDNA Synthesis Kit (Thermo Scientific) following the manufacturer’s instructions. RNA integrity and concentration were verified by spectrophotometry (NanoDrop1000, Thermo Scientific) and 2% agarose gels.

### qPCR

qPCR was performed under the same conditions previously described by Sánchez-Chávez et al. [19]. Primers were design with Primer 3 [20, 21], purchased from T4 Oligo (Irapuato, Guanajuato, Mexico). The sequence of each pair of primers is shown in Table 1. Data were analyzed by following the Livak and Schmittgen method [22] using the 18 gene as a reference. For each sample was determined the expression of the α1–α3 and β GlyR expression, and in parallel the 18S expression (reference gene). As described by Livak and Schmittgen [22], the Ct value obtained for the 18S gene—for each sample—was subtracted from values obtained for each of the GlyR subunits in each sample (∆Ct). Therefore, ∆Ct values obtained for the controls were subtracted from ∆Ct values obtained for the GlyR subunits in each sample (∆∆Ct). The fold-change values (2^−∆∆Ct^) were relative to the control condition.

**Table 1:** Primer sequences

Gene	Sequence (5′–3′)	TM (°C)
Glra1	Forward: GAACGGCAACGTCCTCTACA	67
	Reverse: CCACCCTCATCATCCTTGTGA	
Glra2	Forward: CCTGGGCTAACTGATGGTCC	66
	Reverse: GTGGTTTCTGTGACCGATCC	
Glra3	Forward: TGGCAAGATGAAGCACCAGT	66
	Reverse: GATACCCAACGCTACCCGAG	
Glrb	Forward: TGAGGCAGAAGTGGAACGAC	66
	Reverse: CTCACCAACCTGCAAAGTGC	
18S	Forward: TACCACATCCAAGGAAGGCAGCA	75.6
	Reverse: GCCAGCAAGCCGCGGTAATTCCA	

### Western Blotting

Spinal cord homogenates or synaptosomes were resuspended with lysis RIPA buffer containing proteases and phosphatases inhibitors (Tris–HCl 10 mM, H 7.5, EGTA 2 mM, NaCl 158 mM, Na_2_MoO_4_ 10 mM; NaF 25 mM, EDTA 1 mM, bacitracin 1 mg/ml, benzamidine 2 mM, soybean trypsin inhibitor 0.1 mg/ml, pepstatin 10 μg/ml, aprotinin 1.2 μg/ml, leupeptin 4 μg/ml, Triton X-100 2%, SDS 0.2%) for 1 h at 4 °C under constant shaking. Total protein (30 μg) was loaded in 10% acrylamide gels and run for 2 h at a constant voltage. Afterwards, proteins were transferred to polyvinylidene fluoride (PVDF) membranes, which were blocked (3 h) with 1% albumin-delipidated milk (5%) dissolved in buffer TBS-Tween (Trizma 20 mM, NaCl 136 mM, Tween-20 0.1% pH 7.6). The transference efficiency was corroborated by staining the membranes with Ponceau S solution. Membranes were incubated with the respective primary antibody (anti-α1 GlyR (1:2500; 146,003, Synaptic systems; RRID:AB_2108989); anti-α3 GlyR (1:1000 ab118924, Abcam; RRID:AB_10903015); anti-α2 GlyR (1:1000, ab97628, Abcam; RRID:AB_10680442); anti-GlyRβ (1:2000, ab136239, Abcam; RRID:AB_2939031), and α-actin (1: 2000, ab3280, Abcam; RRID:AB_303668). Later, membranes were incubated for 1 h in the presence of the secondary antibody coupled to horseradish peroxidase (anti-Rabbit-HRP (1: 15,000, NA934, Cytiva; RRID: AB_772206); anti Mouse-HRP (1: 15,000, NA931, Cytiva; RRID: AB_772210)). The signal was visualized with chemiluminescence using the Hyperfilm ECL reagent (Immobilon Western Chemiluminescent HRP Substrate, Millipore Corp.) and digitized with the DigicDoc Rt Alfa software (Alpha INNOTECH). Relative values of each GlyR subunit were normalized with respect to α-actin (Supplementary Fig. S1).

### Statistical Analysis

All data were analyzed with the GraphPad Prism 5 software and statistical significance was determined by the One-way ANOVA analysis, followed by Tukey’s post hoc test.

## Results

### Model of Study

Throughout the investigation, the body weight of the STZ-treated rats was lower, and the blood glucose levels and water intake were three to fourfold higher than the control animals (Table 2) [23].

**Table 2:** Body weight, blood glucose levels and water intake in control and streptozotocin treated rats

	7 days	20 days	45 days
Body weight (g)			
Control	207 ± 27 (5)	307 ± 40 (5)	393 ± 25 (10)
STZ	200 ± 16 (5)	212 ± 15 (9)*	215 ± 12 (7)*
Blood glucose (mg/dl)			
Control	126 ± 16 (11)	142 ± 17 (10)	138 ± 18 (7)
STZ	406 ± 75 (10)*	459 ± 71 (10)*	453 ± 70 (7)*
Water intake (ml/24 h)			
Control	42.3 ± 2.5 (3)	42.7 ± 15 (3)	45.0 ± 8 (3)
STZ	140 ± 17 (3)*	172 ± 21 (3)*	201 ± 14 (3)*

Data are the mean ± SD of at least three different rats per group conducted in triplicated. The sample size (N) is in parenthesis

*p > 0.05

### GlyR Subunits Expression

#### mRNA

**Fig. 1:**
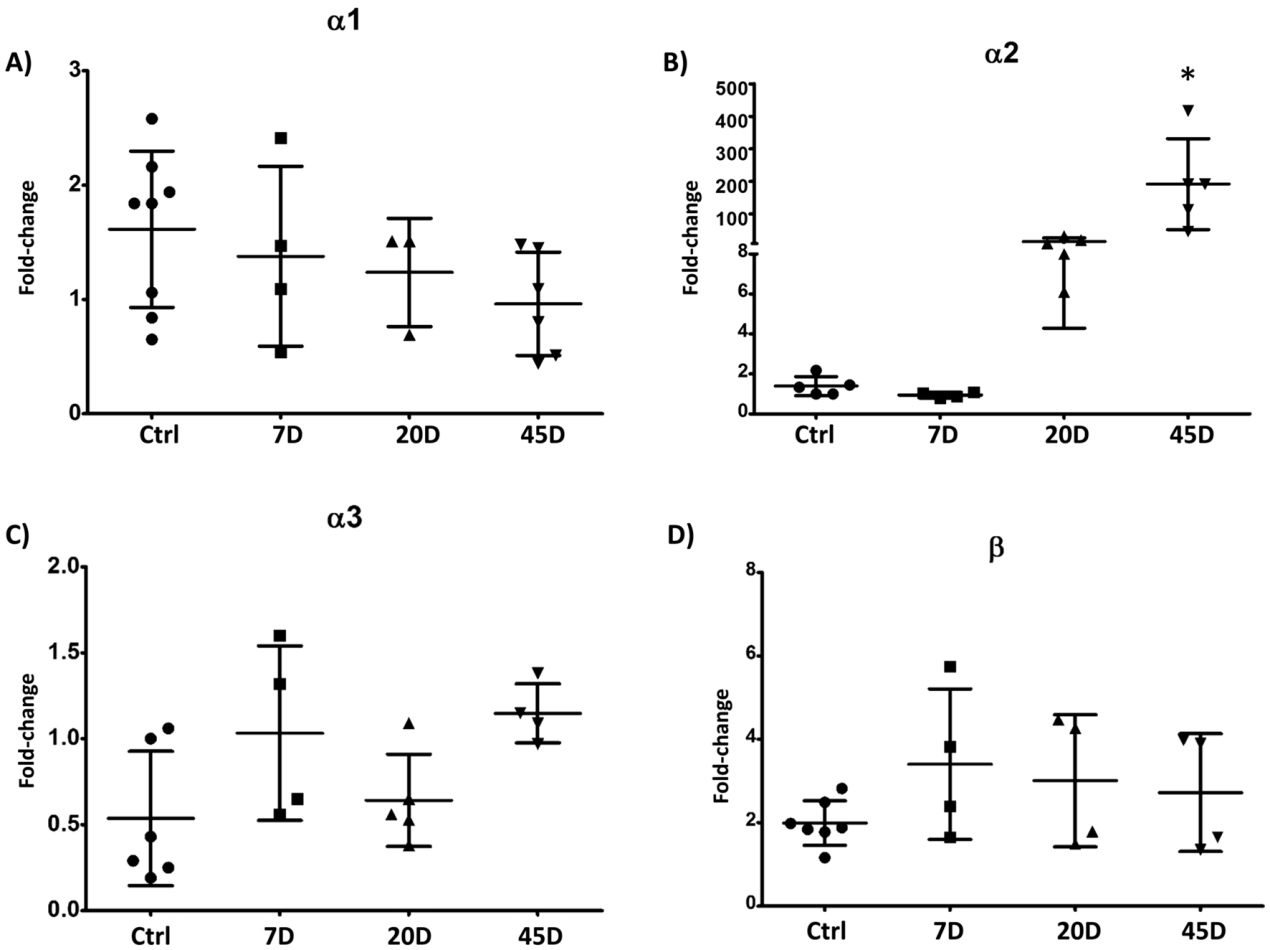
mRNA GlyR expression in the whole spinal cord. The mRNA expression of the GlyR was evaluated by qPCR. Data showed no changes in the α1 (**A**), α3 (**C**), and β (**D**) GlyR subunits, but that of the α2 subunit (**B**) was markedly increased at 45 days. Data were analyzed with Livak and Schmittgen method using the 18 gene as a reference. For each sample was determined the expression of the α1-α3 and β GlyR expression, and in parallel the 18S expression (reference gene). As described by Livak and Schmittgen [22], the Ct value obtained for the 18S gene—for each sample—was subtracted from values obtained for each of the GlyR subunits in each sample (∆Ct). Therefore, ∆Ct values obtained for the controls were subtracted from ∆Ct values obtained for the GlyR subunits in each sample (∆∆Ct). The fold-change values (2^−∆∆Ct^) were relative to the control condition. Data are the mean ± SD of at least three animals per group conducted in triplicated

At mRNA levels, spinal cord GlyR subunits showed expression changes significantly different to that of the non-diabetic. The expression of the α1, α3, and β subunits did not show changes on their expression (Fig. 1A, C, D) and that of the α2 GlyR significantly increased at 45D (control: 1.4 ± 0.48 vs 45D: 192 ± 140) (Fig. 1A, C, D).

#### Protein

**Fig. 2:**
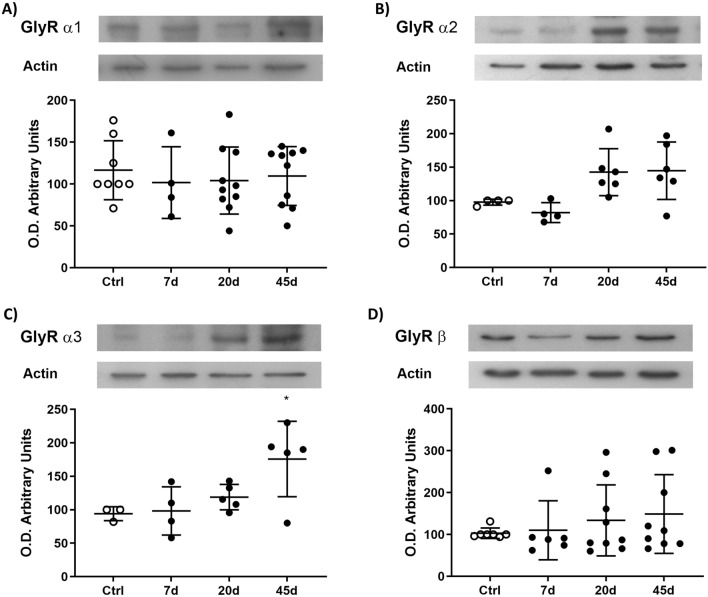
Protein GlyR expression in the whole spinal cord. The α1 (**A**), α2 (**B**), and β (**D**) GlyR subunits did not showed expression changes in any of the studied conditions. However, the expression of the α3 (**C**) GlyR subunit significantly increased at 45 days, respectively. Data are the mean ± SD of at least three different rats per group conducted in triplicated

In the spinal cord, the GlyR subunits protein expression was evaluated in both the cell body and in synapses. In the whole spinal cord homogenates from control animals, the relative protein α3 subunit expression was around 50% higher than the α1 subunit (not shown). In the STZ-treated rats, the α1, α2, and β GlyRs expressions were not statistically different to those observed in the control animals, but the α3 GlyR expression increased at 45D (82 ± 56%) (Fig. 2A–C).

**Fig. 3:**
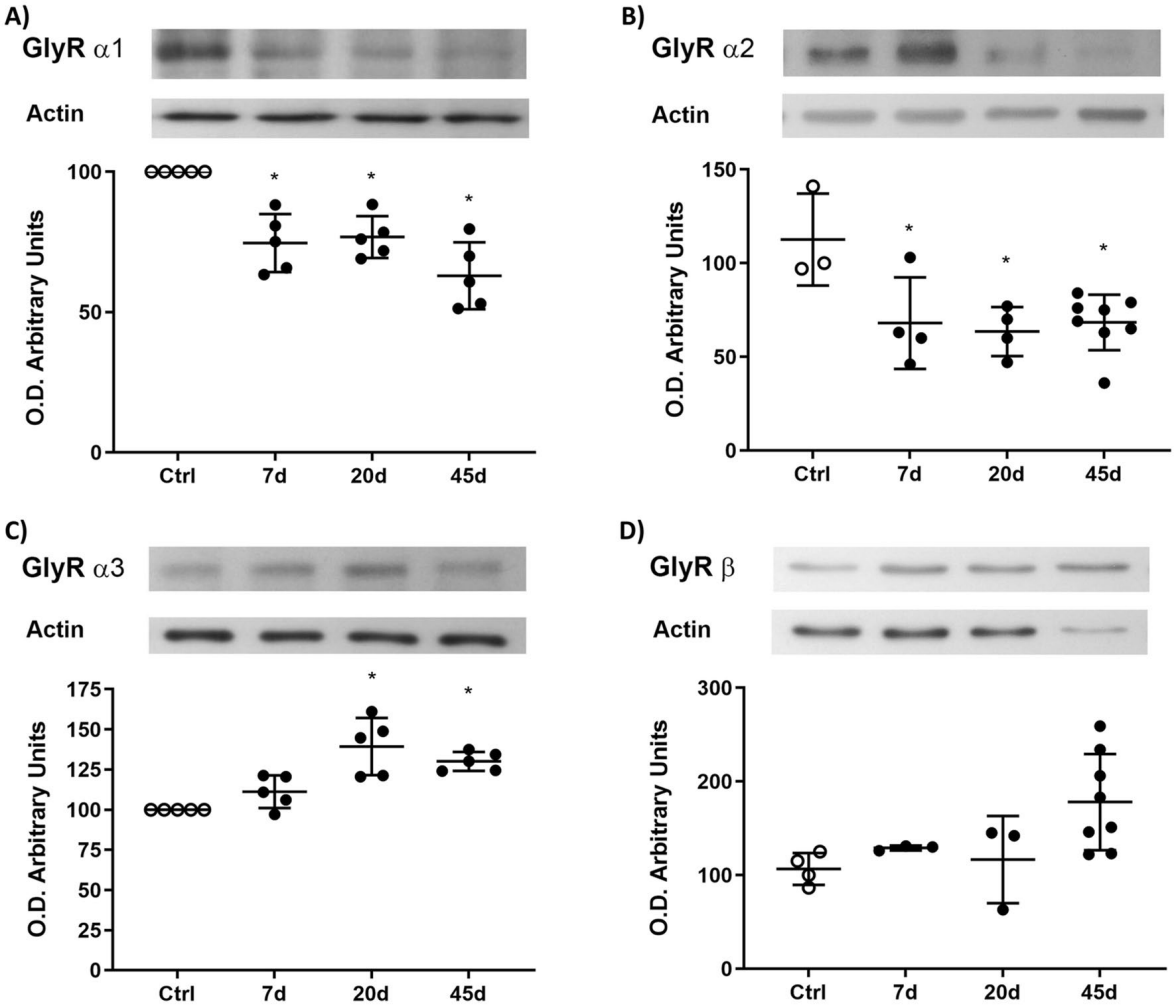
Protein GlyR expression in spinal cord synaptosomes. **A**, **B** The α1 and α2 expression significantly decreased at 7 days, 20 days, and 45 days, but that of the α3 (**C**) subunit increased at 20 days and 45 days. **D** The expression of the β subunit did not show significant changes. Data are the mean ± SD of three rats per group conducted in triplicated

Meanwhile, in synaptosomes, the α1 (around 29%) and α2 (around 33%) GlyR subunits expression statistically decreased on all days of treatment, but that of the α3 subunit increased at 20 (39 ± 18%) and 45 days (30 ± 6%); the β subunit did not show expression changes (Fig. 3A–D).

#### Alterations in Pain Sensitivity in Streptozotocin-Injected Rat

The pain sensitivity in diabetic-induced rats was evaluated through a capsaicin-evoked nocifensive test. While saline stimulus did not cause a significant response in control nor diabetic rats, capsaicin injection produced higher paw-licking behavior in the STZ-induced diabetic rats, but this change was only statistically significant at 45 days compared to the control and 20 days rats (Table 3).

**Table 3:** Licking behavior of control and diabetic rats

	Saline stimulation	Capsaicin stimulation
Control	9.8 ± 5.1 (5)	24 ± 8.3 (5)
Diabetic 20 days	6.6 ± 4.2 (5)	31 ± 15 (10)
Diabetic 45 days	8.4 ± 3.6 (5)	57 ± 26 (10)*

Values represent the cumulative licking time in seconds, measured during 10 min (PLT), and are the mean ± SEM of at least five animals per group

*p ≤ 0.05 respect to the control. The number of determinations are shown in parenthesis

## Discussion

One of the prominent symptoms of diabetic neuropathy is neuropathic pain, which affects 16% of patients with diabetes; however, it is frequently unreported and inadequately treated [24]. The pathogenesis of diabetic neuropathic pain is complex and, thus, remains poorly understood. The involvement of the glycinergic neurotransmission in nociception is supported by the fact that its attenuation increases both hyperalgesia and allodynia [4, 12] and α2 GlyRs attenuate mechanical hyperalgesia induced by zymosan [25]. In STZ-diabetic rats, neuropathic pain was associated with a decrease in the inhibitory action of glycine due to the reduction in its presynaptic release [6]. STZ is the most common agent used to induce experimental type 1 diabetic syndrome in animals, and hyperalgesia might develop within 2–3 weeks [26]; therefore, we analyzed the α1–α3 and β subunits expression in STZ-induced diabetic rat spinal cord.

Both, α1 and α3 GlyRs are in the superficial dorsal horn and co-localizes in around 50% of synapses, indicating that both types of receptors could be acting in a synergic manner to control pain sensitization. As well as by controlling specific nociceptive pathways, as it has been demonstrated for α2 compared to α3 GlyRs [4, 12, 24]. In this respect, we did not find changes in the GlyRs expression in the whole tissue, while significant decrease in the α1 subunit was observed in synaptosomes. The reduction of the α1 subunit in synaptosomes strongly suggests a decrease in the glycinergic neurotransmission and, consequently, the attenuation of the inhibition signal and pain sensitization.

In inflammatory pain, the involvement of α3 GlyRs is relatively well studied [12, 27–29]. Their PKA-dependent phosphorylation in response to Prostaglandin 2 (PGE2) attenuated their function, which in turn increased pain sensitivity [12, 13]. Chiu et al. [6] showed that pain sensitization during diabetes was associated with a decreased pre-synaptic release of glycine instead of the GlyR function. Related to this, Imlach et al. [4] demonstrated that neither α3 GlyRs nor PGE2 were regulating nociceptive pathways after nerve injury, which indicates the involvement of nociceptive pathways independent of the α3 GlyRs activity and dependent of other GlyR subunits, such as α1 and/or α2 [28, 29]. The observed decrease in the α1 subunit expression might support such conclusion.

α2 GlyRs are preferentially expressed at early spinal cord development and their expression is drastically reduced in adults [25, 30, 31]. In the spinal cord, α2 GlyRs were shown to be involved in the attenuation of zymosan-induced hyperalgesia [25] and they were overexpressed after nerve injury [4]. Interestingly, in the whole tissue we found a considerable increase of the total α2 subunit expression at intermediate and late stages of diabetes, but as for the α1, decrease on its expression was observed in the synaptic fraction, which might be explained by a disturbance in the traffic of the protein to the synapses. The latter may also explain the decrease in the post-synaptic α1 GlyRs. Therefore, the decrease of α1 and α2 GlyRs expression at the synapses should lead to hyperalgesia. By contrast, the increase in the α3 GlyRs at post-synapsis might be related to the emergence of an inhibitory system trying to control chronic pain.

It is well known that α2 GlyRs have low decay kinetics compared to α1 or α3 GlyRs [32, 33] and that they inhibit neuronal excitability in a sustained manner in this way. According to this, the decrease in the number of synaptic α1 and α2 GlyRs might be related to a decrease in the inhibitory glycinergic action with the consequent increase in pain levels. Kallenborn-Gerhardt et al. [25] showed that α2 GlyRs could be part of a pain relief system since its expression was attenuating hyperalgesia.

In fact, the STZ-treated rats displayed an increase in pain sensitivity produced by capsaicin, and this sensitization increases according to the days of exposure to STZ (Table 3). These results agree with previous reports where mechanical and thermal sensitivity changes were observed in STZ-treated animals [26, 34, 35]. These observations strengthen the point that the glycinergic inhibitory neurotransmission decreased in this model of diabetic neuropathy.

## Conclusions

Our results indicate changes in the expression pattern of GlyR subunits in early stages of STZ-induced hyperglycemia, suggesting a key role of these receptors on neuropathic pain.

### Supplementary Information

Below is the link to the electronic supplementary material.

Supplementary file1 (JPG 7064 KB)
**Supplementary Figure S1** Representative Western blots for the expression of GlyR subunits from rat lumbar spinal cord. The weight of each subunit is indicated by an arrow. The expression of each of the GlyR subunits was normalized *versus* actin expression. The actin gel for each condition is at the bottom. MW: Molecular weight. N1: Control animal 1; N2: Control animal 2; 7d1/d2: STZ-treated independent animals (7 days). 20d1/d2: STZ-treated animals (20 days); 45d1/d2: STZ-treated animals (45 days). The band selected for analysis was marked with a rectangle (dotted line).

Supplementary file2 (JPG 4687 KB)
**Supplementary Figure S2** Representative SDS-PAGE electrophoresis gels and Western Blotting of the GlyR subunits expression from rat lumbar spinal cord synaptosomes. Actin was used as loading control. (dotted line). N: Control non-treated animals; 7d: STZ-treated animal (7 days); 20d: STZ-treated animals (20 days); and 45d: STZ-treated animal (45 days). The band selected for analysis was marked with a rectangle.

## Data Availability

The datasets generated during the current study are available from the corresponding author on reasonable request.
